# Teacher competence and students’ motivation for learning in Chinese higher education: mediating roles of psychological flourishing and student engagement, and the moderating role of AI integration

**DOI:** 10.1186/s40359-025-03674-0

**Published:** 2025-12-29

**Authors:** Xiang Chen, Sarminah Samad, Wansoo Kim

**Affiliations:** 1 School of Teacher Education, East China Normal University, Shanghai, 200062 China; 2 Shaoxing University, Zhejiang, 312000 China; 3 Department of Management, College of Business Administration, Princess Nourah bint Abdulrahman University, Riyadh, 11671 Saudi Arabia; 4 Gachon University, Seongnam-si, Republic of Korea

**Keywords:** English learning motivation, Teacher competence, Student engagement, AI integration, Flourishing in learning

## Abstract

This study explores how teacher competence influences university students’ English learning motivation in China, through the mediating roles of students’ psychological flourishing and engagement, and whether integrating AI in teaching moderates these effects. Guided by self-determination theory (SDT), we hypothesized that competent teachers foster an environment that enhances students’ well-being and active involvement, which in turn sustains higher motivation. Survey data were collected from *N* = 467 undergraduates studying English in Chinese higher education, and analyzed using partial least squares structural equation modeling (PLS-SEM). The results confirmed that teacher competence positively predicts students’ flourishing in English learning (a state of optimal well-being in the learning context) and student engagement (active participation in learning) (both *p* < 0.001). In turn, both flourishing and engagement show significant positive effects on students’ English learning motivation (*p* < 0.001). The mediation analysis revealed that these psychological and behavioral factors significantly carry the influence of teacher competence to motivation. Moreover, AI integration in teaching (e.g. using AI tutoring/chatbot support) moderated the pathway from teacher competence to student outcomes: at higher levels of AI use, the positive effects on engagement and motivation were amplified (interaction *p* < 0.01). These findings demonstrate an integrated framework where effective teaching and supportive technology jointly promote student motivation, extending SDT to a technology-enhanced context. The study underscores the importance of teacher training for competence, pedagogical strategies focused on student engagement and well-being, and culturally responsive AI-enhanced learning tools. Such a comprehensive approach can sustain and enrich students’ motivation for English learning in Chinese higher education.

**Supplementary Information:**

The online version contains supplementary material available at 10.1186/s40359-025-03674-0.

## What is already known

- Teachers with strong pedagogical competence, including cognitive, affective, and psychomotor skills, significantly boost students’ motivation to learn English by fostering autonomy, relatedness, and a supportive classroom environment.
  EFL learners who experience higher levels of positive emotions, meaning, and engagement (i.e., psychological flourishing) demonstrate stronger academic motivation and persistence, as well as greater willingness to communicate in English.

- AI-powered applications (e.g., adaptive platforms, chatbots) have been shown to increase behavioral, cognitive, and emotional engagement among EFL learners, which in turn translates into higher motivation and reduced procrastination.

## What this study contributes

- By combining teacher competence, learners’ psychological flourishing, and AI-enabled resources within one framework, this study addresses the lack of research examining all three predictors together, particularly among Chinese EFL learners.
- Structural equation models confirm that engagement (behavioral, emotional, cognitive) fully mediates how teacher competence, flourishing, and AI tools influence English learning motivation, underscoring the central role of engagement.
- Grounded in empirical findings, the study provides context-specific strategies, such as professional development for teachers, positive‐psychology interventions for learners, and scalable AI platforms, to holistically enhance motivation in Chinese EFL settings.

## Introduction

The global language position of English is becoming increasingly dominant due to its dominant role in international communication, business, academia, and technology [1]. English language has changed from a privilege to an essential requirement for contemporary employment and higher education, since it opens professional opportunities and international partnerships [2]. Thus, learning English is at the forefront of education systems all over the world. In China, a country that is playing an increasing role in global markets and international relations, English skills are especially important [3]. Despite many years of formal English learning, Chinese students are often weak in terms of fluency and functional communication. This calls for the need to better understand the factors that contribute to successful English learning in this context, beyond the conventional language teaching [4].

One of the main difficulties associated with English language learning, particularly in English as a Foreign Language (EFL) setting, is maintaining the motivation of the students to learn English where there is no possibility for real-life immersion. Recent studies emphasize that motivation does not develop in a vacuum; instead, teachers play a central role in meeting students’ autonomy, competence and relatedness needs [5]. In China, these relationships are complicated by cultural norms rooted in Confucian heritage. Teachers are often regarded as unquestionable authority figures, and students typically defer to them [6]. Such hierarchical expectations can inhibit students from voicing their needs or challenging pedagogical practices. Recognizing these cultural dynamics is essential when applying SDT to Chinese classrooms. This study situates teacher competence within this cultural context, examining whether autonomy-supportive practices can still fulfil students’ psychological needs and thus sustain intrinsic motivation, engagement and flourishing.

Student motivation can be described as “the degree to which a person makes efforts or strives to learn the language through desire to do so and the satisfaction derived in the process” [7]. Highly motivated learners tend to stay for longer, learn more deeply and reach higher proficiency in the target language [8]. Previous studies have shown that teachers are instrumental to fostering this motivation. Highly qualified teachers, who have high pedagogical competence, content knowledge, and classroom skills, provide the right atmosphere that meets the needs of students and makes them more interested in the learning process [9]. For example, students’ motivation and academic achievement are higher when they perceive their teacher as competent and supportive [10]. In EFL classes, in which learners are usually very dependent on the teacher, the role of teacher competence is even more important in order to have learner engagement. Experiential: Learners need to experience a competent English teacher’s use of interactive methods, providing clear feedback and linking lessons to their lives to develop the intrinsic motivation and confidence of the students [11]. According to self-determination theory (SDT), such pedagogy can fulfill learners’ basic psychological needs (e.g. feeling capable and supported), resulting in higher self-intrinsic motivation [12].

Besides the role of teachers, the psychological well-being and interest of students in learning as factors contributing to their motivation have been identified [13, 14]. Psychological flourishing is a concept that represents the well-being, positive emotions and the sense of meaning of the learning process to a student. EFL students who thrive, with increased positive affect and purpose with regard to their education, are more prone to greater persistence and academic drive [15]. When students are happy, confident and their learning of the English language has a purpose, there is a tendency that they will make effort and keep on enhancing their skills in the language. Another important aspect is student engagement, which implies the level of attention, interest, and active participation shown by students in the learning process [16, 17]. Students with high levels of engagement are more active in the classroom, do not hesitate to train language, and do not lose concentration even when the task is difficult. Engagement has a strong connection with motivation: the more behaviorally and emotionally engaged learners in the learning process, the more intrinsic motivation they tend to develop because they feel more competent and in control of their learning process [18]. In fact, the roles of engagement can serve as a medium between helpful teaching and student motivation; when teachers engage students by encouraging their motivation tend to increase as well as their performance.

In conjunction with pedagogical and psychological aspects, the new educational technology has turned out to be a new frontier that affects student motivation. Artificial intelligence (AI) applications and platforms (incl. intelligent tutoring systems, adaptive learning applications, and chatbots) are finding more and more uses in language education [19]. Such AI-based tools have the potential to adapt the learning content, give instant feedback, and offer interactive practice, which, in turn, has been found to enhance student engagement and decrease learning anxiety [20]. According to early research, it is possible to note that AI integration can enhance the motivation of EFL learners and make the process more independent and focused on specific needs [21]. As an example, AI chatbots and intelligent teaching systems can help maintain students in language-related tasks at a higher level of cognitive and emotional engagement, which will support their desire to continue. But what is still unclear is how AI can affect or regulate other human-filled factors (such as the influence of a teacher or the psychological condition of a student). The vast majority of the technology-in-education studies focused on the functional advantages of AI (e.g. automation, content delivery), but the interaction between AI and motivational psychology and instructional practice is not explored thoroughly yet.

### Research gaps and objectives

Hitherto, very few studies have conducted a holistic examination of how teacher characteristics, student psychological thriving, student engagement and technology interact to influence language learning motivation. Most of the previous studies have investigated these aspects separately [22, 23], e.g. studying teacher competence in general education classrooms, or evaluated AI tools without taking psychological mediators into account [24]. Moreover, a large portion of the literature on English learning motivation is based in the Western setting [25]. Little is known about the working of these dynamics in higher education settings in East Asia, including China, where culturally informed expectations and classroom rules might be different. AI-assisted learning tools promise personalized feedback and adaptive content. However, recent research shows that large language models such as ChatGPT 3.5 exhibit cultural stereotypes and biases; for example, ChatGPT 3.5 displayed significant cultural bias on decision-making tasks, while ChatGPT 4.0 reduced but did not eliminate these biases [26, 27]. Scholars have called for technical oversight and transparent training data to mitigate such biases [28]. This study therefore considers technological support not only as an enabler of learning but also as a potential source of cultural bias. We discuss how culturally diverse AI systems might influence learners’ motivation and well-being.

Also, despite flourishing being a topic that has attracted some attention in the educational research, its mediating role on teacher competence and motivation in the EFL context has not been fully investigated, as the literature currently concentrates on general education (Chaves, 2021). Third, engagement of students, an important indicator of academic success [29, 30], is not sufficiently studied as a mediating variable of teacher competence and motivation in EFL learning. Likewise, although the topic of AI integration has received more attention [31], there has been little research on the influence of AI on teacher-student relationships and psychological variables, such as motivation and flourishing (English learning flourishing from now onward).

This research fills these gaps by considering the combination of pedagogical, psychological, and technological factors in a single framework. On the basis of self-determination theory, we hypothesize that teacher competence must positively affect student motivation through direct and indirect effects via positive influence on student English learning flourishing and engagement, and that AI integration in educational practice will only increase the positive effects of English learning flourishing and engagement on motivation. In short, the major aim of the study is to explore the connection between the English teacher competence and English motivation of learners through the lenses of the underlying mechanism (English learning flourishing and engagement as mediators) and one of the major contextual determinants (AI integration as moderator). Our context is Chinese EFL learners in universities and thus the motivation theory is examined into an under-studied area in literature. In doing this, we also wish to point out the importance of teacher competence and student well-being in enhancing language performance and to indicate how the use of AI technology can be used to enhance them. Finally, the paper provides some insights that are not only applicable to China but also to other audiences worldwide who might be interested in how best to integrate effective teaching, positive psychology, and AI innovation to help language learners become more motivated.

### Theoretical framework (self-determination theory)

This research is theoretically grounded in SDT, which provides a lens for understanding how teacher behaviors, student psychological states, and technology converge to influence motivation. SDT posits that individuals have basic psychological needs for autonomy, competence, and relatedness, and that when these needs are satisfied, intrinsic motivation and well-being are enhanced [32]. SDT distinguishes between intrinsic and extrinsic motivation along a continuum of autonomy [5]. When teachers provide autonomy support, structure and interpersonal involvement, learners’ basic psychological needs are satisfied. In language learning contexts, autonomy-supportive teaching predicts higher self-efficacy, engagement and performance. Conversely, controlling practices undermine motivation and engagement. This study extends SDT by investigating how teacher competence (knowledge, pedagogical ability and relational skills), students’ psychological well-being, learning engagement and technological support jointly influence English learning flourishing in English learning motivation. Drawing on positive psychology, flourishing is conceptualized as a holistic state encompassing positive emotion, engagement, relationships, meaning and accomplishment [33].

To improve clarity, we define the core constructs used in our model. Teacher competence refers to teachers’ pedagogical knowledge, subject expertise and interpersonal skills. Psychological well-being denotes students’ overall mental health and positive affect, aligning with positive psychology’s view that flourishing entails more than the absence of distress [34]. Engagement refers to behavioral, emotional and cognitive involvement in learning activities [5]. We use the term learning engagement throughout to avoid confusion with other forms of engagement. Flourishing in English learning is defined as students’ experience of positive emotions, sustained motivation, a sense of meaning and accomplishment in language learning [35]. Technological support encompasses digital tools and AI-based resources that facilitate learning. Flourishing in English learning (psychological well-being) and student engagement (behavioral involvement) are related but conceptually distinct. Flourishing reflects an inner sense of thriving [36], while engagement represents active learning behavior [37]. We included both as mediators to capture complementary aspects of students’ experiences. Although flourishing and engagement can influence each other, flourishing students tend to engage more, and engagement can enhance well-being; our model treats them as parallel pathways from teacher input to motivation to highlight their unique effects. For theoretical clarity and parsimony, we retained them as independent mediators.

**Fig. 1:**
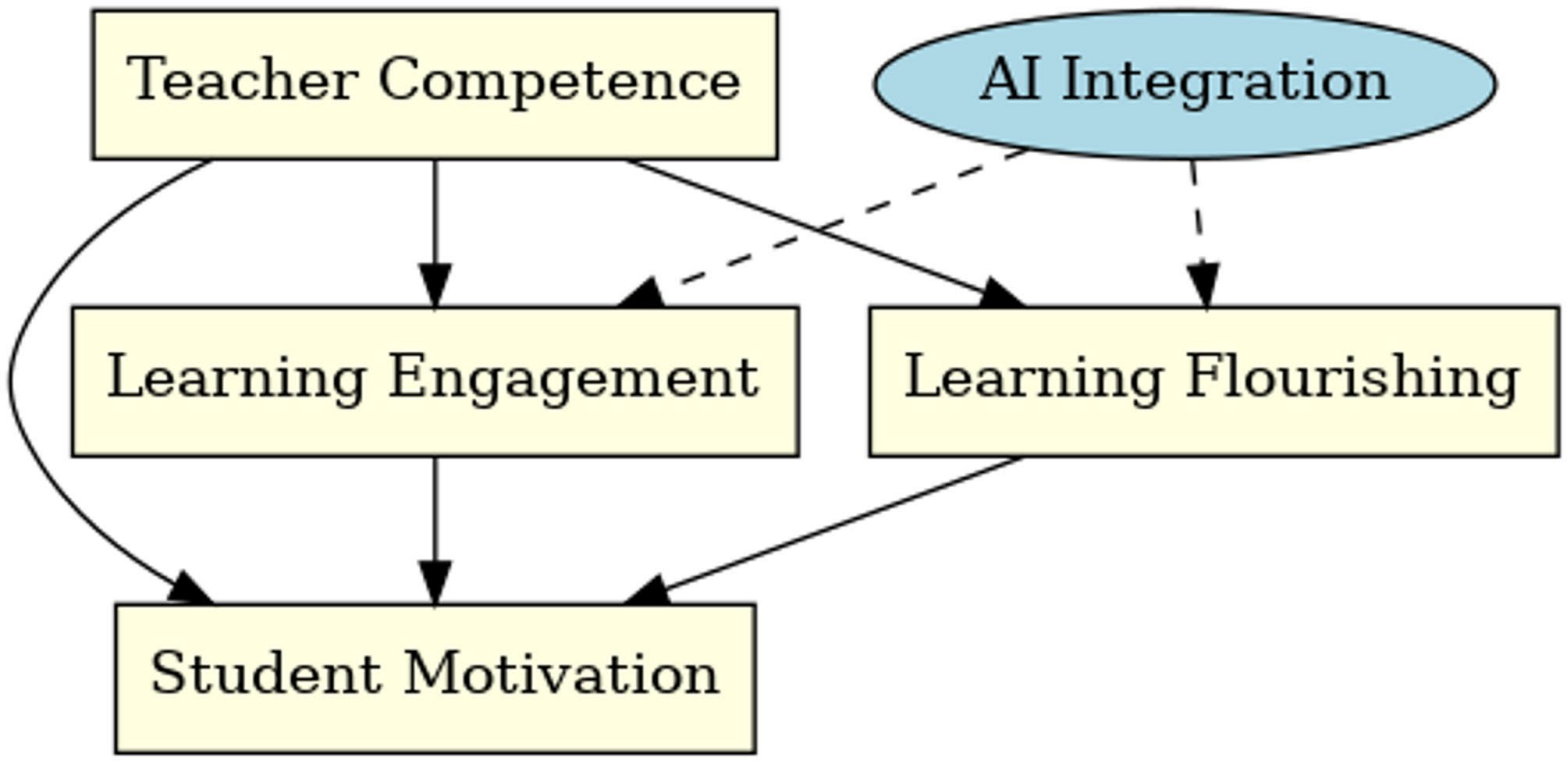
Conceptual framework depicting how teacher competence influences student English learning motivation directly and indirectly via students’ English learning flourishing and engagement, with AI integration moderating the pathways from English learning flourishing and engagement to motivation. Solid lines represent hypothesized direct or mediating effects, and the dashed lines represent the moderating effects of AI

In an educational context, a competent and supportive teacher can fulfill students’ need for competence (by helping them feel capable in mastering English) and relatedness (by building a positive teacher-student rapport), thereby nurturing higher motivation [9]. Students’ English learning flourishing and engagement are both indicative of these needs being met, and in turn, they fuel greater intrinsic motivation. For example, when a student feels enjoyment and accomplishment in English learning (flourishing) and is deeply engaged in class activities, it suggests that the learning environment is satisfying their needs, leading to sustained self-driven effort. We also theorize that AI integration can play a facilitative role in this process. AI tools, by offering personalized practice and immediate feedback, can support students’ sense of competence and autonomy in learning [17]. As a result, AI may amplify the positive effects of English learning flourishing and engagement on motivation, essentially acting as a catalyst that strengthens how psychological well-being and active learning translate into motivational gains. In summary, SDT ties together our model’s elements: teacher competence acts as an external support that satisfies fundamental needs; this in turn elevates students’ English learning flourishing and engagement (mediators), which directly boost motivation; and AI functions as a modern resource that enhances these relationships. This theoretical framework guides our hypotheses and interpretation of results, ensuring our investigation is anchored in established motivational theory while exploring novel interactions (see Fig. 1 for the conceptual model).

## Literature and hypotheses development

### The relationship between English teacher competence and student English learning motivation

Teacher competence can be defined as the knowledge, skills and abilities of teachers to be able to make students learn and attain the desired academic results [9]. It includes subject-matter competence, instructional effectiveness, classroom organization, and establishing positive relationships with the students. Regarding language teaching, a well-prepared EFL teacher is able to modify learning in accordance to student needs, employ interactive techniques, and provide a favorable atmosphere, thus directly affecting the attitude and motivation of the students to learn. Student English learning motivation on the other hand refers to the motivation, need, and intent that leads to a student learning English [7]. It consists of intrinsic motives (e.g. learning English because it is pleasant in its own right) and extrinsic motives (e.g. learning English because I will be able to achieve some career or academic rewards). Motivation is essential as it promotes, directs and sustains language learning behavior in the long term - more highly motivated students put more effort into it, persist, and attain superior proficiency levels in the English language [38].

The Chinese educational context is distinctive in its hierarchical classroom norms and the high value placed on teacher authority [6]. Such norms may suppress students’ autonomy and thus undermine intrinsic motivation. Studies of Chinese vocational students show that teacher–student interactions are often authoritative and that students feel powerless to challenge teachers. These cultural dynamics may moderate the effects of teacher competence on psychological well-being and engagement. Our model therefore accounts for cultural specificity by examining whether autonomy-supportive teacher competence can overcome hierarchical norms and satisfy students’ psychological needs. Previous research has established the existence of a positive correlation between the competence of the teacher and student motivation. Students tend to feel motivated and confident in learning when they feel that their teacher is competent, well-organized, and helpful [39]. Lauermann and ten Hagen [22] discovered that teachers who are highly perceived competent by students are able to boost the motivation and academic interest of said students in any subject. This effect is frequently described in social-cognitive and self-determination terms: effective instruction meets psychological needs of students in competence (to help them pass learning tasks) and relatedness (with nurturing student-teacher relationship) and, thus, promotes internal motivation [40]. Teacher competence is particularly very important in EFL contexts because learners rely heavily on instruction and support [41]. Constructive feedback, interactive learning and student-centered approaches are important in motivation [42]. Being able to see teachers as knowledgeable and helpful enhances intrinsic motivation and persistence of learners. SDT suggests that effective teaching satisfies the students need of autonomy, competence and relatedness, which fosters long-term motivation [43]. Based on such insights, we anticipate that:

- H1 English teacher competence is positively associated with student English learning motivation.

### Teacher competence, student English learning motivation, and the role of English learning flourishing

Flourishing is conceptualized within positive psychology as optimal functioning characterized by positive emotions, deep engagement, supportive relationships, purposeful meaning and a sense of accomplishment [33]. This PERMA model posits that each domain contributes uniquely to well-being and can be pursued for its own sake. When students experience English learning flourishing, they are more resilient, self-efficacious and motivated to learn English. Flourishing, a key component of positive psychology, refers to an individual’s sense of well-being, purpose, and engagement in life [44]. In educational settings, English learning flourishing is closely linked to students’ psychological development, learning satisfaction, and academic achievement [45]. Teachers play a central role in fostering English learning flourishing by creating a learning environment that supports their emotional and cognitive needs [46]. When students feel valued, capable, and encouraged by their teachers, they are more likely to develop a strong sense of purpose and engagement in their academic journey, ultimately enhancing their overall well-being [47]. The competency level of EFL teachers plays a major role in how students experience English learning flourishing in their learning process. The teachers’ competence in English brings meaningful growth to students through their structured instruction interactive teaching and individualized feedback [48]. Through precise teaching methods, supportive criticism, and motivational guidance teachers enable students to build language confidence that creates both a feeling of success and maintained interest. Students who experience higher English learning flourishing levels during their English learning demonstrate enhanced persistence enjoy the learning process better and achieve better long-term results [49].

The state of flourishing defines how much students become motivated to learn in education. Those students who find psychological fulfillment and well-being in their learning demonstrate increased intrinsic motivation because they understand that academic achievements lead to personal development and achievement as indicated by Fink [50]. Student desire for ongoing education strengthens because positive emotions develop across engagement self-worth and confidence. A classroom environment that provides support and enrichment leads students to achieve higher flourishing levels that drive their academic persistence through enthusiasm toward their goals [51]. In English learning, English learning flourishing directly contributes to students’ motivation. When EFL learners experience psychological fulfillment in their learning process, they develop intrinsic motivation to improve their skills. Feeling competent in language use, supported by peers and teachers, and emotionally engaged in learning activities increases students’ drive to master English [52].

Building on SDT, English learning flourishing fulfills students’ psychological needs, particularly competence, and relatedness, which strengthen their intrinsic motivation [53]. Teacher competence fosters English learning flourishing by creating an environment where students feel supported and valued. In turn, English learning flourishing strengthens students’ internal drive to learn English. Thus, English learning flourishing serves as a mediating mechanism in the relationship between teacher competence and student motivation. Because English learning flourishing satisfies students’ psychological needs for competence, relatedness and autonomy, it may mediate the link between teacher competence and motivation. Based on this discussion, the following hypotheses are proposed:

- H2 English teacher competence is positively associated with English learning flourishing.
- H3 English learning flourishing is positively associated with student English learning motivation.
- H4 English learning flourishing mediates the relationship between English teacher competence and student English learning motivation.

### Teacher competence, student English learning motivation, and the role of English learning engagement

Student engagement is generally defined as the degree of attention, interest, curiosity, and active involvement that a student exhibits in the learning process [17]. It encompasses behavioral engagement (participation in academic activities), cognitive engagement (investment in learning and self-regulation), and emotional engagement (enthusiasm and positive emotional connection to learning). Engaged learners tend to be attentive in class, put effort into homework, contribute to discussions, and persist even when facing challenges, which often correlates with better learning outcomes. This concept is closely tied to motivation: when students are engaged, it is both a sign of underlying motivation and a mechanism that can further enhance motivation by providing a sense of accomplishment and autonomy.

Academic success together with student retention and educational quality depends directly on the level of student engagement [54]. Student engagement shows itself through their learning activities as well as mental focus and emotional dedication toward academic work [55]. Teachers serve as essential support for student engagement through their creation of interactive learning spaces that provide support as well as stimulation to actively participate in their studies [56]. Students become more willing to participate in class content through the use of teaching methods which include group work and practical examples and immediate feedback [57]. The acquisition of English language as second foreign language depends on sustained effort and continuous practice and interaction because language acquisition requires these elements according to Susanti [58]. English teachers who are competent develop student engagement through language activities combined with thinking skills and flexible classrooms designed to accommodate different ways students learn [59]. Students who think their teachers offer support and preparation while being motivational will participate actively in language learning activities and practice English outside the classroom and keep up their desire to master the language [60]. When teachers motivate students through active involvement English learning becomes more important to them which results in better language mastery.

Student motivation shows strong associations with engagement according to Senior, Bartholomew [61]. Student participation in their studies leads to intrinsic motivation because students feel they have control and satisfaction during their academic work. Guests who participate in learning experiences develop intensified motivation through beneficial feedback that enhances their confidence and prevents them from experiencing burnout according to Paloș, Maricuţoiu [62]. Active student participation in language learning leads to better English skill development which produces higher motivation through persistence and resilience and enthusiasm [63]. The psychological needs of autonomy competence and relatedness that students require are met through engagement according to SDT while creating stronger intrinsic motivation for English learning [64]. Students become more engaged through teacher-provided meaningful instruction which enables enhanced student learning activities while developing English language emotional engagement. The relationship between teacher competence and motivation gets strengthened through engagement which serves as a vital mediating factor. The discussion leads to the development of the following research hypotheses:

- H5 English teacher competence is positively associated with English learning engagement.
- H6 English learning engagement is positively associated with student English learning motivation.
- H7 English learning engagement mediates the relationship between English teacher competence and student English learning motivation.

### The moderating role of AI integration in English education

Modern educational systems benefit from AI innovation since it develops innovative approaches to support educational development [65]. The educational tools powered by AI include chatbots and personalized tutoring systems as well as automated feedback mechanisms which provide students with immediate help while creating adjustable learning pathways and practice materials [66]. Research demonstrates how these technologies boost learner commitment while improving their learning speed together with their motivational levels through individualized educational support which goes beyond conventional classroom activities [67]. AI integration creates the most beneficial effects when teachers use it to enhance their instruction and thus allow students to experience increased learning through technological methods [68].

AI tools for English language learning create valuable resources which enhance student learning by providing interactive exercises and speech assistance and automatic feedback for writing and grammar [69]. The correct integration of AI tools strengthens teacher achievements which leads to increased English learning motivation and confidence among students [70]. Student reliance on AI tools beyond moderation along with treating them as substitutes for teacher contact results in decreased engagement and lowered intrinsic motivation [39]. AI integration in English education will affect student motivation through English learning flourishing and engagement based on its implementation strategies in the learning process. The implementation of AI technology within education either strengthens the psychological needs of students or creates obstacles for their development according to SDT principles [71]. AI tools that enable student autonomy and competence strengthen their intrinsic motivation according to Chiu, Moorhouse [67]. However, excessive reliance on AI may reduce meaningful teacher-student interactions, potentially weakening motivation. Thus, this study examines AI integration as a moderating factor in the relationship between English learning flourishing, engagement, and motivation. While numerous studies have explored AI’s general impact on learning (e.g., adaptive learning systems improving performance) [24], there is limited research examining how AI tools might moderate the relationships between traditional factors (like engagement or well-being) and motivation. In other words, it remains unclear whether technology can amplify the benefits of an engaged, English learning flourishing student on his/her motivation to learn, a question this study seeks to answer. Based on this discussion, the following hypotheses are proposed:

- H8 AI integration in English education moderates the relationship between English learning flourishing and student English learning motivation, such that the relationship is stronger when AI integration is high.
- H9 AI integration in English education moderates the relationship between English learning engagement and student English learning motivation, such that the relationship is stronger when AI integration is high.

## Methodology

### Participants and data collection procedure

University students enrolled in EFL courses in four Chinese cities, Nanjing, Wuhan, Xi’an, and Hangzhou, participated in this study. These cities were chosen for their diverse institutions and emphasis on English learning. Structured questionnaires were distributed in person by research assistants across classrooms and language labs in three waves, each about four weeks apart, ensuring representation and reducing response bias. In each of these cities, we requested several universities to allow us to interview students taking mandatory courses in College English. Intact EFL classes were chosen within each university through cluster sampling (i.e. all students within the chosen class were invited to take part) to sample a wide variety of learners. It is a pragmatic and yet diverse strategy to accommodate students with varying majors and year of study. It is also consistent with educational research advocacy to employ multi-site samples to enhance generalizability. The final sample contained 467 responding valid responses (after filtering out those that were incomplete), and demographic information is summarized in Table 1. The information was obtained by using paper-and-pencil questionnaires that were conducted by trained research assistants during the classes sessions in three waves separated by about four weeks. This time-lagged (i.e. three-wave) design was used to minimize common method bias [72, 73] and to be sure that students had enough time to think about their answers, yet the design remained cross-sectional (all measures were taken during each wave and summed). Participation was voluntary and the respondents were assured of anonymity and confidentiality [74]. No financial incentives were provided to the respondents. The total response rate was approximately 69% (483 returned out of 700 mailed), and 467 surveys were kept after the elimination of unusable responses (e.g., too many missing data, or inattentive response patterns). This is larger than the target sample size of about 400 calculated using power analysis (see 3.3 Sample Size Estimation), which has sufficient statistical power to base our analyses on.

**Table 1:** Participant demographics (*N* = 467)

Variable	Frequency	Percentage
Gender – Male	212	45.4%
Gender – Female	255	54.6%
Age Group – 18–20 years	198	42.4%
Age Group – 21–23 years	205	43.9%
Age Group – 24 years and above	64	13.7%
Year of Study – First Year	132	28.3%
Year of Study – Second Year	158	33.8%
Year of Study – Third Year	106	22.7%
Year of Study – Fourth Year and above	71	15.2%
Primary English Learning Method – Formal courses	311	66.6%
Primary English Learning Method – Online learning	78	16.7%
Primary English Learning Method – Self-study	56	12.0%
Primary English Learning Method – Other	22	4.7%

### Ethical considerations and sample size estimation

This study conformed to the Declaration of Helsinki and was approved by the Shaoxing University IRB (Ref. SU-IRB-EDU-2024-248, 03 Oct 2024). Participant data were anonymized, stored securely, and handled per institutional and national guidelines [75–77]. Participants provided informed consent after being briefed on study objectives, confidentiality, anonymity, and their right to withdraw. Data was securely stored with access limited to authorized researchers.

Using GPower (power = 0.80, significance level = 0.05), the recommended sample size was approximately 400 participants. Considering potential low response rates, 700 questionnaires were distributed. Out of 483 returned, 467 valid questionnaires were retained after removing incomplete or inattentive responses.

### Instrumentation

Each survey instrument was based on validated scales in the literature and contextualized to the Chinese EFL students. Sample items and sources were already available to each construct. We stress that the definitions of each of the construct (which were used to develop our instruments) were the following: Student motivation was assessed in terms of intrinsic enjoyment and internal/external control in studying English (the degree of their desire to learn and the direction of this desire). English learning flourishing was characterized by the academic well-being of students, their positive feelings and motivation to learn English. Engagement was characterized by the active participation of the students in learning (attention, effort and participation) at behavioral, cognitive and emotional levels. The competence of teachers was determined according to the student point of view, i.e. the ability of a teacher to teach and help students in their learning process. The integration of AI was viewed in terms of the degree to which AI-based resources were utilized to assist the student in learning English (e.g. AI chat bots or platforms that help the student practice).

In order to minimize the possibility of item overlap among constructs to establish content validity we made a number of steps. Two professionals in the field of applied linguistics and one in the field of educational psychology reviewed the first pool of items in the questionnaire [78, 79]. They checked the semantic redundancy of any of the items of varying scales or too closely related (which may confuse them or inflate correlation between constructs). During this review, a few items were reformulated to further differentiate between constructs. We also performed a little pilot test on 40 students (a representative sample of the population) to obtain feedback on the difficulty of items and to conduct a preliminary factor analysis. This resulted in a few words being refined and the establishment that each of the sets of items clustered as it should. Subsequently, the final survey was identified with separate sets of items per construct and no apparent overlaps in phrasing and meaning. The consistency of each scale within the pilot was satisfactory (Cronbach’s alpha >0.70 across all constructs), and also in the main study. We used Likert scale ranging from 1 = Strongly disagree to 5 = Strongly agree.

Student English learning motivation was measured using a 12-item scale capturing intrinsic motivation, introjected regulation, and external regulation. Intrinsic motivation reflects the inherent enjoyment of learning English (e.g., “Learning English is enjoyable for me.”); introjected regulation indicates internal pressures to engage (e.g., “I want to prove to my teachers that I can improve my English.”); and external regulation relates to external expectations (e.g., “My family and teachers encourage me to learn English.”). These items were adapted from Ardasheva, Tong [80]. English learning flourishing was assessed with eight items adapted from a flourishing scale [81], evaluating students’ well-being and sense of purpose (e.g., “Learning English gives me a sense of purpose and meaning.”).

English teacher competence was evaluated using five items from a teacher competency scale, measuring how effectively teachers support learning (e.g., “My English teacher connects new lessons with previous ones to help me understand better.”). These items were extracted from the study of Hanaysha, Shriedeh [82]. English learning engagement was measured with five items adapted from Handelsman, Briggs [83], capturing active engagement both inside and outside the classroom (e.g., “I find ways to connect English learning to my daily life.”). AI integration in English education was examined using four items borrowed from the study of Stöhr, Ou [84], assessing how AI-powered tools, especially chatbots, enhance English learning (e.g., “The AI chatbots I use help me understand and communicate English concepts better.”).

Procedural safeguards (anonymity, confidentiality, attention checks) and statistical tests (Harman’s single-factor test, marker variable analysis) confirmed minimal method bias, ensuring robust and valid analysis.

### Data analysis

Since the model is complex and involves mediators, moderators, and latent constructs, we used Partial Least Squares Structural Equation Modeling (PLS-SEM) in SmartPLS as PLS-SEM functions best with non-normal data, exploratory, prediction oriented models and interaction effects [85, 86]. In line with best practices, we have first appraised the measurement model then the structural model. In the case of the measurement model, the reliability of the indicators was ensured because all loadings are above 0.60. The composite reliability was above the 0.70 for all variables [87, 88]. The AVE values were all >0.50. These findings were in favor of convergent validity [89, 90]. Fornell-Larcker and HTMT criteria were also used to confirm discriminant validity. In structural model, the hypothesized relationships were tested by bootstrapping (5,000 resamples) to get the standard errors, t-statistics and 95% confidence interval [91, 92]. The multicollinearity was not a problem (all VIF < 3). We have reported both R^2^ values of explained variance and f^2^ effect size of each predictor. Moderation (H8, H9) was also examined by specifying interaction terms between mean-centered variables, and significant terms were then subject to investigation using simple slope plots to demonstrate the effect of flourishing or engagement on motivation in high vs. low AI integration. It is further to note that, as a variance-based method, PLS-SEM does not report traditional covariance-based fit indices (CFI, TLI, RMSEA). Instead, model fit was assessed via the Standardized Root Mean Square Residual (SRMR = 0.068 < 0.08), showing good fit, and the Normed Fit Index (NFI = 0.907), indicating acceptable model performance. All hypotheses were tested using bias-corrected bootstrapping with 5,000 resamples, and significance of direct, indirect, and moderating effects was determined from 95% confidence intervals excluding zero and corresponding p-values.

These results are reported in Sect. 4 with details.

## Results

### Measurement model evaluation

Tables 1 and 2 present a comprehensive summary of the demographic characteristics, factor loadings, composite reliability (CR), and average variance extracted (AVE). The demographic analysis revealed a balanced gender distribution, with 45.4% male and 54.6% female participants. A vast majority (86.3%) of the respondents were aged between 18 and 23, which is typical for a university student population. Students from all academic levels were included, with second-year students forming the largest group (33.8%). Regarding English learning methods, formal English courses were the primary mode (66.6%), followed by online learning (16.7%), self-study (12.0%), and other methods (4.7%).

**Fig. 2:**
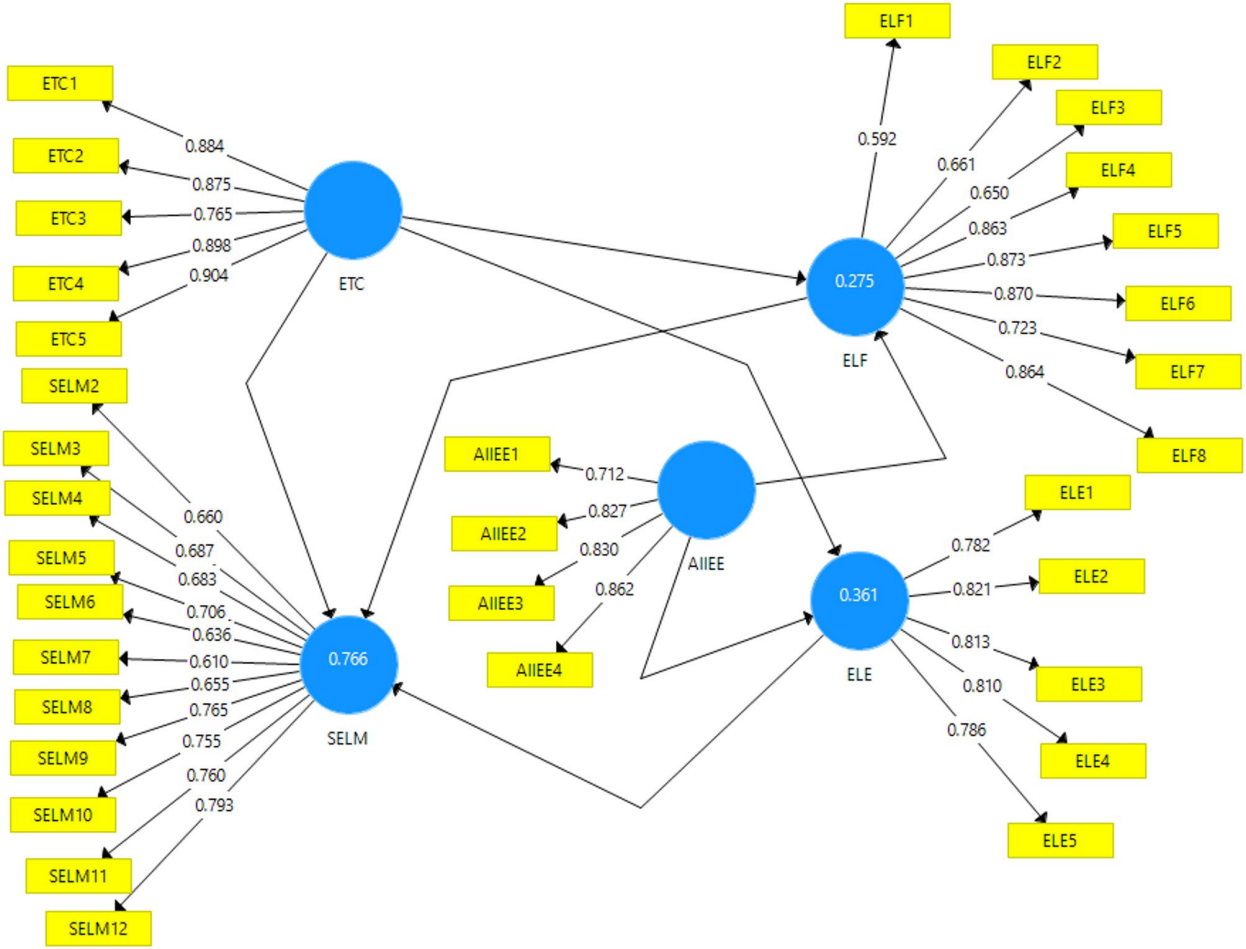
The measurement model

The measurement model was rigorously evaluated by examining the factor loadings of each item, ensuring that all met or exceeded the recommended threshold of 0.6. Specifically, the factor loadings ranged from 0.712 to 0.862 for AI integration in English education (AIIEE); from 0.782 to 0.821 for English learning engagement (ELE); from 0.592 to 0.864 for English learning flourishing (ELF); from 0.765 to 0.904 for English teacher competence (ETC); and from 0.61 to 0.793 for student English learning motivation (SELM). Composite reliability (CR) values for all constructs were above 0.80, confirming strong internal consistency. Moreover, the AVE values, ranging from 0.495 to 0.751, indicate an acceptable level of convergent validity [93, 94]. Overall, these results confirm that the measurement model is robust and well-suited for hypothesis testing. Figure 2 reflects the figurative illustration of our measurement model.

**Table 2:** Measurement model results

Construct	Items	Factor loadings range	AVE	CR
AI Integration in English Education (AIIEE)	4	0.712–0.862	0.655	0.883
English Learning Engagement (ELE)	5	0.782–0.821	0.644	0.901
Flourishing in English Learning (ELF)	8	0.592–0.864	0.593	0.919
English Teacher Competence (ETC)	5	0.765–0.904	0.751	0.938
Student English Learning Motivation (SELM)	12	0.610–0.793	0.495	0.914

*AIIEE* AI Integration in English education, *ELE* English learning engagement, *ELF* English learning flourishing, *ETC* English teacher competence, *SELM* Student English learning motivation, *AVE* Average variance extracted, *CR* Composite reliability

Discriminant validity was evaluated using the Fornell-Larcker criterion and the Heterotrait-Monotrait (HTMT) ratio. Table 3 displays the square root of the AVE along the diagonal, with off-diagonal values representing inter-construct correlations. According to the Fornell-Larcker criterion, the square root of AVE for each construct should exceed its correlations with others [95, 96]. The results confirm this; for instance, AIIEE has a square root of AVE of 0.81, which is higher than its correlations with ELE at 0.417 and ELF at 0.443. Similarly, SELM shows a square root of AVE of 0.703, surpassing its correlations with other constructs. The HTMT ratio further confirmed discriminant validity. With acceptable values below 0.85 (and issues typically arising above 0.90), all HTMT values in Table 3 fall below the threshold. For example, the HTMT ratio between ELE and ELF is 0.727, and between AIIEE and English Teacher Competence (ETC) is 0.418, both well within the acceptable range.

**Table 3:** Discriminant validity analysis

Construct	Square Root of AVE	AIIEE Correlation	ELE Correlation	ELF Correlation	ETC Correlation	HTMT AIIEE	HTMT ELE	HTMT ELF	HTMT ETC
AIIEE	0.81	-	0.417	0.443	0.362	-	0.616	0.522	0.418
ELE	0.803	-	-	0.373	0.403	-	-	0.727	0.53
ELF	0.77	-	-	-	0.422	-	-	-	0.46
ETC	0.867	-	-	-	-	-	-	-	-
SELM	0.703	-	-	-	0.369	0.536	0.666	0.637	0.595

*AIIEE* AI Integration in English education, *ELE* English learning engagement, *ELF* English learning flourishing, *ETC* English teacher competence, *SELM* Student English learning motivation

### Structural model and hypothesis testing

The structural model results are summarized in Table 4; Fig. 2, following the order of our hypotheses.

**Table 4:** Hypothesis testing, R-Square, and F-Square

Construct	Path Coefficient	Standard Deviation	T Statistics	*P* Values	2.5% CI	97.5% CI	*R* Square	f Square
ETC ->SELM (H1)	0.408	0.05	8.181	0	0.299	0.502	-	0.544
ETC ->ELF (H2)	0.3	0.052	5.757	0	0.208	0.404	-	0.109
ELF ->SELM (H3)	0.417	0.048	8.745	0	0.317	0.517	0.275	0.296
ETC ->ELF ->SELM (H4)	0.125	0.025	4.915	0	0.081	0.178	-	-
ETC ->ELE (H5)	0.327	0.048	6.851	0	0.242	0.423	0.361	0.147
ELE ->SELM (H6)	0.225	0.042	5.331	0	0.145	0.304	-	0.081
ETC ->ELE ->SELM (H7)	0.074	0.017	4.222	0	0.044	0.112	0.412	-
Moderating Effect 1 (AIIEE) ->ELF ->SELM (H8)	0.066	0.021	3.092	0.002	0.023	0.092	-	0.134
Moderating Effect 2 (AIIEE) ->ELE ->SELM (H9)	0.048	0.012	3.857	0	0.08	0.059	-	0.215

*AIIEE* AI Integration in English education, *ELE* English learning engagement, *ELF* English learning flourishing, *ETC* English teacher competence, *SELM* Student English learning motivation

#### Direct effects

ETC was hypothesized to directly influence SELM, ELF, and ELE, while ELF and ELE were expected to predict motivation (H1, H2, H5, H3, H6). PLS-SEM results supported all five hypotheses. ETC significantly enhanced SELM (H1: β = 0.408, t = 8.181, *p* < 0.001), ELF (H2: β = 0.300, t = 5.757, *p* < 0.001), and SELM (H5: β = 0.327, t = 6.851, *p* < 0.001). ELF (H3: β = 0.417, t = 8.745, *p* < 0.001) and ELE (H6: β = 0.225, t = 5.331, *p* < 0.001) each positively influenced SELM. These results confirm that ETC and the mediators each play a significant role in shaping SELM.

#### Mediating effects

We proposed that ELF and ELE mediate the impact of ETCon SELM (H4, H7). Bootstrapped analyses supported both pathways. ELF mediated the effect of ETC on SELM (H4: indirect β = 0.125, t = 4.915, *p* < 0.001), while ELE also mediated this link (H7: indirect β = 0.074, t = 4.222, *p* < 0.001). Both mediations were partial, as the direct effect (H1) remained significant. ELF exerted a somewhat stronger mediating influence than ELE, but both are critical mechanisms through which ETC fosters SELM.

#### Moderating effects

AI integration was hypothesized to strengthen the links between ELF and SELM (H8) and between ELE and SELM (H9). Both moderations were significant: ELF × AI (β = 0.066, t = 3.092, *p* = 0.002) and ELE × AI (β = 0.048, t = 3.857, *p* < 0.001). Simple slopes showed steeper positive effects of ELF and ELE on SELM at higher levels of AIIEE, confirming that AI tools amplify the benefits of psychological well-being and active learning on motivation.

#### Explained variance and effect sizes

**Fig. 3:**
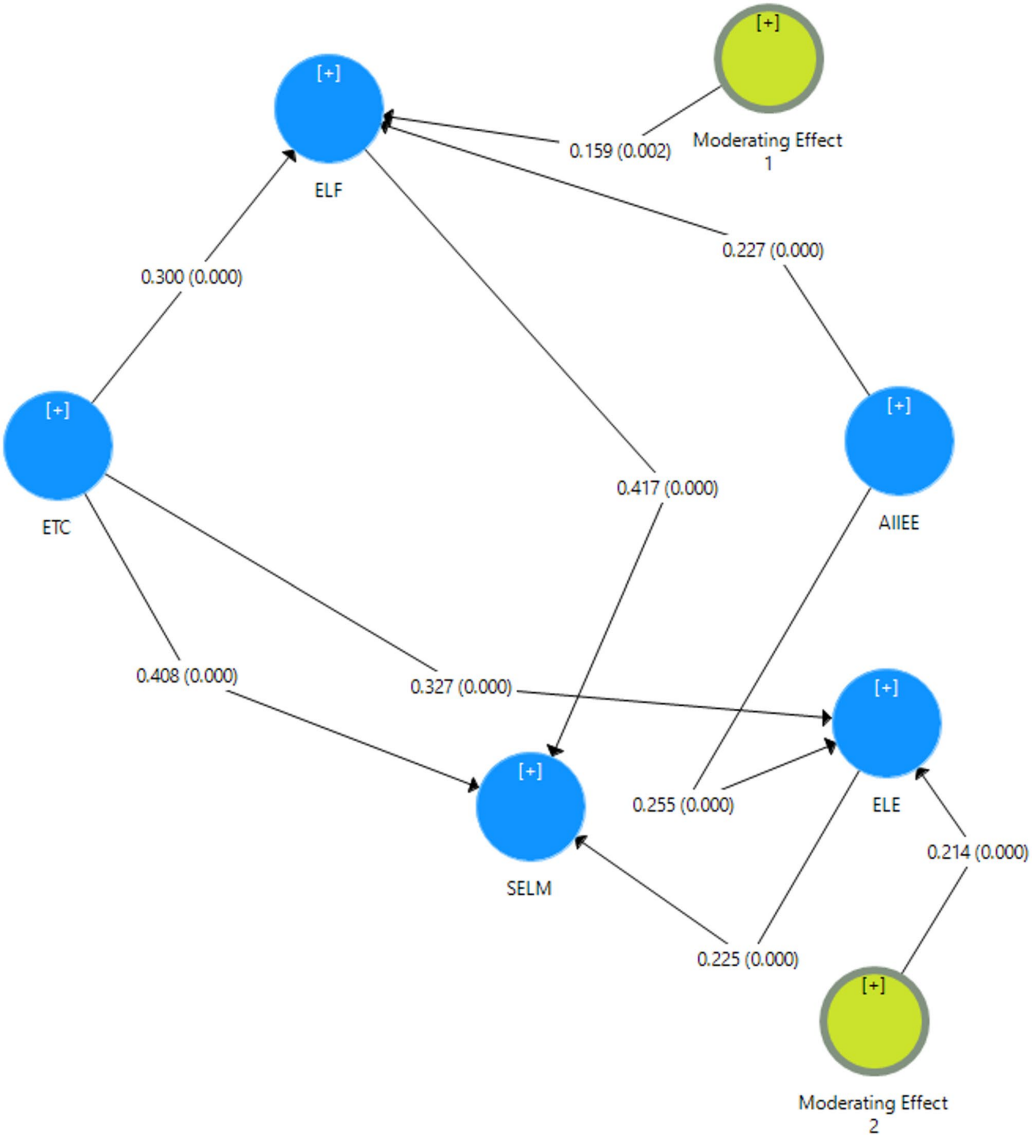
The structural model

ETC explained 27.5% of the variance in ELF (R² = 0.275) and 36.1% in ELE (R² = 0.361). The full model accounted for 41.2% of the variance in motivation. Effect size analyses showed ETC had a large direct effect on SELM (f² = 0.544), with flourishing exerting a medium-to-large effect (f² = 0.296). Effects of ETC on ELF (f² = 0.109) and ELE (f² = 0.147) were smaller but meaningful. The AIIEE effects were medium in size (f² = 0.134 and 0.215). All nine hypotheses (H1–H9) were supported. Figure 3 presents our structural model.

## Discussion

This research validates the importance of teacher competence as a vital factor for motivating students in language learning, echoing numerous prior studies in general education. Consistent with our results for H1, capable and supportive teachers directly boost students’ motivation to learn English, confirming that effective teaching practices (e.g. clear instruction, engaging activities, helpful feedback) inspire greater learner interest and effort [11, 97]. This finding reinforces well-established educational psychology principles: for example, teachers who fulfill students’ needs for competence and relatedness create a classroom climate where students want to engage (as suggested by SDT). Our study extends this knowledge by demonstrating that teacher competence not only has a direct impact but also indirectly affects motivation through English learning flourishing and engagement. Earlier research had hinted at these pathways. For instance, Jelas, Azman [98]and others observed that engaged students often emerge in classrooms with high-quality teaching, and those engaged students tend to be more motivated. Moreover, the hierarchical teacher–student relationships prevalent in Chinese classrooms may limit students’ autonomy. Our results suggest that when teachers adopt autonomy-supportive practices, encouraging student voice, providing rationale and showing care, students’ basic psychological needs can still be met, enhancing well-being and engagement despite cultural constraints. Consequently, teacher training in need-supportive pedagogy is essential to foster intrinsic motivation.

We were able to empirically confirm both mediation routes in an EFL context: when students feel they are “flourishing” (experiencing positive well-being) and are actively engaged, these states carry the influence of teacher competence forward to enhance motivation. Students show greater intrinsic motivation when they find learning meaningful according to previous studies [45, 46]. This investigation verifies that competent English teachers make their students flourish which results in substantial increases in their English learning motivation. This study improves upon existing literature since it establishes an English learning context for flourishing while making new contributions. The research confirms previous studies about students maintaining better academic performance when they feel more accomplished in their learning activities and display improved well-being [54]. This result is an important addition to the literature, because it integrates positive psychology (flourishing) and engagement research into the discussion of teacher effects on language learning. It suggests that one mechanism for how great teachers motivate students is by making their students happier and more engaged learners, not just by transmitting knowledge. Students who feel supported by a competent teacher are more likely to enjoy learning English and find it meaningful, thereby developing intrinsic motivation to continue improving. They are also drawn into participating and investing effort, which builds a self-sustaining cycle of motivation and success. The findings of this study build upon previous research [22, 41] and confirm that English teacher competence demonstrates direct motivation effects on student English learning but also creates indirect effects via English learning flourishing and English learning engagement. Our findings are in line with SDT’s propositions: the teacher, as a social context factor, can satisfy students’ psychological needs; satisfied needs lead to flourishing (a sense of growth and well-being) and engagement (active involvement), which in turn yield higher quality motivation.

Although, technological support positively influenced engagement and flourishing. However, AI systems are not culturally neutral; ChatGPT 3.5 exhibited cultural stereotypes and significant bias in decision-making tasks, while ChatGPT 4.0 reduced but did not eliminate these biases. These findings caution against uncritical adoption of AI tools. Educators should select AI systems that are transparent about their training data and bias mitigation strategies. Integrating culturally diverse AI models and using prompt strategies can help reduce biases. Future research should compare the motivational impact of different AI models across cultures and evaluate whether culturally sensitive AI promotes more equitable learning experiences.

One of the most novel findings of this study is the moderating role of AI integration. We found that incorporating AI tools into English learning significantly strengthened the positive effects of both English learning flourishing and engagement on student motivation. In simpler terms, a motivated mental state (English learning flourishing) and active learning behavior (engagement) translated into even greater motivation when students also had AI-based support in their learning process. This result differentiates our work from previous studies that examined technology or AI in isolation [65]. Earlier research on AI in education showed that AI-driven platforms can improve engagement or learning outcomes generally [17], but it seldom looked at *for whom* or *under what conditions* AI provides the most benefit. Our moderated moderation analysis indicates that AI is especially beneficial for students who are already psychologically invested in learning, it amplifies their motivation. This could be because AI tools, such as intelligent tutoring systems or chatbots, offer *personalized challenges*,* instant feedback*,* and adaptive learning pathways* that keep enthusiastic students even more engaged and help flourishing students channel their positive energy into concrete progress [17]. From an SDT perspective, AI might be supplying additional autonomy (students can learn at their own pace) and competence feedback (immediate knowledge of results), which boosts the motivational impact of an engaged, need-satisfied student. Notably, our focus on psychological outcomes goes beyond the functional view of AI; we demonstrate that AI’s value in education includes *psychological advantages* – it can serve as a catalyst for motivation by augmenting human-centered factors. This insight contributes to emerging discussions on “EdTech” by highlighting that technology’s effects are not uniform for all students; they depend on students’ internal states. In practice, it implies that combining positive education (ensuring students flourish and engage) with AI tools yields a synergistic effect on motivation. To our knowledge, this is one of the first studies to empirically show such an interaction in an EFL context, marking a unique contribution of our work.

Student English learning motivation develops through direct and indirect pathways when English teachers demonstrate their competence according to SDT’s principles. The research confirms the theory and shows that student’ intrinsic motivation grows when their psychological needs of competence and relatedness receive fulfillment. The combination of English teacher competence as well as English learning flourishing and English learning engagement satisfies students’ psychological needs for competence and relatedness. The application of SDT in English language learning receives additional support from AI integration which strengthens motivational effects in the learning process.

Our findings support SDT by demonstrating that teacher competence fulfilling students’ autonomy, competence and relatedness needs fosters psychological well-being and engagement [5]. When students perceive their teachers as knowledgeable, supportive and empathetic, they report higher well-being and engage more deeply in learning activities. Engagement, in turn, predicts English learning flourishing, confirming positive psychology’s assertion that engagement is a core component of flourishing [35]. The strong mediation pathways highlight the importance of nurturing psychological well-being and engagement to sustain intrinsic motivation in English learning.

### Theoretical implications

Our work has some theoretical contributions. First, we combine SDT with the context of EFL and AI to build-up the knowledge base on the application of the classic motivational principles in the technology-enabled language learning. Earlier studies have tended to consider teacher influence, student well-being, and educational technology independently; we put forward and confirm a more holistic model in which the three interact to build motivation. We demonstrated that teacher competence is not only a direct motivator (as extensively reported) but also an indirect one by creating conditions of English learning flourishing (fulfilling the psychological needs of students) and engagement. Such a multi-pathway impact highlights that teacher competence plays dual role-instructional and psychological- in motivating learners. Second, we introduce the construct of English learning flourishing to the discourse of language learning motivation. Although flourishing (or student well-being) has been successfully used in positive education research, few studies have investigated it as an intervening factor in the context of second language acquisition. Theoretical arguments that emotional well-being can underlie academic motivation are supported by our evidence that flourishing is strongly associated with teacher support and motivation. It implies that fostering positive emotions and a sense of achievement in language students is not only a feel-good objective but is also capable of having a positive effect on their motivational paths. Third, and within the same vein, we re-establish the dominant role of engagement in the motivation model and provide some nuance by defining it as the most important behavioral pathway by which teaching influences motivation. This shows the importance of engagement in linking external support (teacher, technology) with internal motivation - an engaged student feels a sense of autonomy and competence in action, which SDT identifies as a key attribute of intrinsic motivation. Lastly, we introduce AI integration as a contextual moderator, which puts the development of theory in a new direction of including technological context in theories of motivation. Our results support the idea that the traditional motivation theories (such as SDT) can be supplemented with technological variables; in this way, it was found that the presence of AI may alter psychological mechanisms (such as the impact of engagement on motivation). This brings new theoretical issues concerning the interactions of digital tools with human motivation - a field that could be pursued further. Overall, our research adds a more comprehensive theoretical framework that more accurately represents the current pedagogical conditions in which educators, the psychology of students, and technology jointly affect the learning outcome.

### Practical implications

The study reveals important implications that educators along with institutions and policymakers need to use for improving EFL student motivation levels. Strong teacher competence directly affects student motivation so institutions should fund teacher training which develops effective teaching strategies and student-centered educational methods along with skills for promoting student engagement and well-being. Professional development initiatives should remain active because they prepare teachers to establish supportive classrooms while delivering recommendations for progress and implementing innovative approaches suitable for students’ psychological appetite and motivational growth.

The research shows that maintaining English learning flourishing is essential to maintaining student motivation levels. Teachers should transition from traditional educational approaches into an environment that makes language education seem meaningful and purposeful. Connectivity between classroom instruction and real-life uses of English together with concrete demonstrations of practical skill value can help students reach their goals. Student language-learning success depends on the promotion of teamwork with autonomy alongside self-evaluation practices that allow them to develop meaningful purpose in their language development. Research indicates that student motivation and teacher competence have English learning engagement as their essential connection. Teachers should implement interactive teaching methods such as problem-based learning and role-playing as well as discussions that actively engage students to boost engagement. Students’ interest receives additional support through digital tools coupled with gamification elements and interactive assessments above institutional commitments to student-driven learning initiatives which support peer collaboration and active participation.

AI integration functions as a moderator that indicates technology should be employed to increase motivational levels. AI systems together with chatbots and intelligent tutors deliver customized learning that gives real-time assessment and interactive activities to boost student engagement and confidence levels. Effective training of educators to use AI tools demands learning how to implement these tools so they help students learn without eliminating human teaching presence. Additionally training students in AI platform usage and providing guidelines for their AI platform access is equally important. The growing presence of AI in education warrants higher education institutions to build strategic direction for AI integration into teaching programs. Public authorities must back research projects that evaluate AI-enhanced educational settings regarding their influence on student drive and academic success measurements. Government education policies must focus on enhancing instructor capabilities by implementing digital competency instruction with mental well-being methods and participation strategies in teacher training curricula. These recommendations enable educators and institutions alongside policymakers to establish better English learning methods that develop student motivation and maintain their well-being while teaching essential skills necessary for academic and professional achievement.

Although this study was conducted in China, the insights are broadly relevant to English learning contexts worldwide. The interplay of teacher quality, student mental states, and technology is a global concern. In many countries, educators face similar challenges: how to keep students motivated to learn a second language in an era of distractions and diverse learner needs. Our research suggests a multifaceted solution, invest in teachers, nurture student well-being, and intelligently deploy technology. For example, in Western contexts where autonomy is emphasized, ensuring teachers also provide the supportive structure (competence and relatedness support) could further boost intrinsic motivation. In developing countries where technology is less prevalent, policymakers might focus first on teacher training and engagement strategies, while planning for gradual integration of ed-tech tools. Conversely, in highly digitized education systems, there may be a tendency to adopt the latest AI tools without sufficient attention to pedagogy or student psychology; our findings caution that technology should not be a standalone fix but part of a larger motivational ecosystem. Cross-cultural generalizability of our findings is likely, given that SDT’s core needs (autonomy, competence, relatedness) are considered universal. Still, we encourage educators to consider cultural nuances: for instance, Chinese students may respond strongly to teacher competence because of culturally high respect for teachers, whereas in some cultures peer influences might also be significant. Nonetheless, ensuring teachers are well-equipped and students are happy and engaged learners is a universally sound strategy. Education policymakers internationally should also note the evidence that technology can enhance learning outcomes when combined with positive human factors; thus, funding decisions should pair investments in digital infrastructure with training programs that help teachers and students use these tools in motivationally optimal ways. In sum, our study’s implications – strengthen teacher capacity, foster English learning flourishing/engagement, and embrace AI as a supportive tool – can inform policy and practice across different educational contexts to improve language learning motivation on a global scale.

### Limitations and future research directions

Despite its contributions, this study has some limitations. First, it was conducted within Chinese higher education, which may restrict the generalizability of the findings to other cultural and educational settings. Future research should extend the study to different countries or systems to examine if similar relationships exist elsewhere. Second, the reliance on self-reported data may introduce social desirability bias; incorporating objective measures or longitudinal designs could better capture changes in student motivation over time. Another limitation is that while the study identified English learning flourishing and engagement as mediators, it did not explore other potential psychological and behavioral mediators such as self-efficacy or cognitive engagement. Future research could expand the model by including these additional variables for a more comprehensive understanding of motivational processes in English learning. Additionally, although AI integration was examined as a moderator, other moderating factors, like institutional support, peer collaboration, or digital literacy skills, merit exploration. Finally, the cross-sectional design limits causal inference. Employing experimental or longitudinal methodologies in future research would offer clearer insights into the causal pathways influencing student English learning motivation.

## Conclusion

This study provides valuable insights into the factors influencing student English learning motivation within Chinese higher education. By satisfying students’ autonomy, competence and relatedness needs, competent teachers promote well-being and engagement. Engagement, in turn, catalyzes English learning flourishing. Technological support enhances engagement and flourishing but should be adopted critically given emerging evidence of AI bias. Practical implications include the need for professional development programs that cultivate autonomy-supportive teaching and for institutional investment in culturally sensitive AI tools. Policymakers should also consider cultural dynamics when implementing AI-assisted language education. Future research could explore comparative effects of different AI models and longitudinal changes in motivation and well-being.

By applying SDT, the study shows that fulfilling students’ needs for competence, relatedness, and autonomy through supportive teaching, engagement, and technological tools strengthens motivation. These insights contribute to our theoretical understanding of motivation in EFL learning and offer practical recommendations for educators, institutions, and policymakers. Moving forward, addressing the study’s limitations through longitudinal research and expanding investigations to diverse educational settings will further enhance our understanding of student motivation. By promoting competent teaching, active engagement, and AI-supported learning, institutions can foster more effective and motivating environments for EFL students.

## Supplementary Information

Supplementary Material 1.

## Data Availability

De-identified data and syntax files are available from the corresponding author on reasonable request.

## Abbreviations

AI: Artificial intelligence
AIIEE: AI integration in English education
EFL: English as a foreign language
ELE: English learning engagement
ELF: English learning flourishing
ETC: English teacher competence
IRB: Institutional Review Board
PLS: SEM–Partial least–squares structural–equation modelling
SDT: Self–Determination Theory
SELM: Student English learning motivation
