# Supplementary material for: Teacher competence and students’ motivation for learning in Chinese higher education: mediating roles of psychological flourishing and student engagement, and the moderating role of AI integration
Source: BMC Psychol. 2025 Dec 29;13:1378. doi: 10.1186/s40359-025-03674-0 (PMC12752287; doi:10.1186/s40359-025-03674-0)
Supplement: Supplementary file 1 — Supplementary Material 1. [file 40359_2025_3674_MOESM1_ESM.docx]

**Questionnaire items**

| **Variable** | **Item No.** | **Items** |
| --- | --- | --- |
| **Student English Learning Motivation** | 1 | Learning English is enjoyable for me. |
|  | 2 | I like discovering new things through English. |
|  | 3 | I enjoy learning about English-speaking cultures and lifestyles. |
|  | 4 | I feel happy when I perform well in English. |
|  | 5 | I feel proud when I understand difficult concepts in English. |
|  | 6 | I enjoy challenging myself with difficult English tasks. |
|  | 7 | I would feel disappointed if I couldn't communicate in English at school. |
|  | 8 | I would feel uncomfortable if I couldn't speak English with my international friends. |
|  | 9 | I want to prove to my teachers that I can improve my English. |
|  | 10 | I am learning English because it will help me get a good job in the future. |
|  | 11 | My family and teachers encourage me to learn English. |
|  | 12 | English is important because everyone needs to learn it. |
| **English Learning Flourishing** | 13 | Learning English gives me a sense of purpose and meaning. |
|  | 14 | My relationships with teachers and classmates support my English learning. |
|  | 15 | I am engaged and interested in improving my English skills. |
|  | 16 | I actively help and encourage others in their English learning journey. |
|  | 17 | I feel confident and capable in using English for important tasks. |
|  | 18 | I am a dedicated English learner and strive to improve my skills. |
|  | 19 | I am optimistic about my future because of my ability to learn English. |
|  | 20 | People respect me for my efforts in learning English. |
| **English Teacher Competence** | 21 | My English teacher connects new lessons with previous ones to help me understand better. |
|  | 22 | My English teacher highlights the key points to improve my learning. |
|  | 23 | My English teacher clearly defines the topics that will be covered in the next lesson. |
|  | 24 | My English teacher provides practical examples to explain theoretical concepts in English learning. |
|  | 25 | My English teacher uses eye contact and gestures to encourage my participation in class. |
| **English Learning Engagement** | 26 | I find ways to connect English learning to my daily life. |
|  | 27 | I apply what I learn in English classes to real-life situations. |
|  | 28 | I explore different ways to make learning English more engaging and enjoyable. |
|  | 29 | I think about and practice English between class sessions. |
|  | 30 | I am eager to improve my English skills and learn more about the language. |
| **AI Integration in English Education** | 31 | The AI chatbots I use make me more effective in learning English. |
|  | 32 | The AI chatbots I use help me understand and communicate English concepts better. |
|  | 33 | The responses generated by AI chatbots often improve my English learning compared to studying alone. |
|  | 34 | Using AI chatbots helps improve my English proficiency and academic performance. |
